# Osborne’s ligament: Anatomical study with application to better understanding ulnar nerve compression at the elbow

**DOI:** 10.1007/s10143-026-04307-9

**Published:** 2026-04-28

**Authors:** Emily M. Persons, Brianna L. Hines, Marcela Herrera, Emma R. Lesser, Muralidharan Anbalagan, Mitesh Dave, Samir Anadkat, Georgi P. Georgiev, Aaron S. Dumont, Joe Iwanaga, R. Shane Tubbs

**Affiliations:** 1 Tulane University School of Medicine, New Orleans, LA USA; 2 Department of Structural and Cellular Biology, Tulane University School of Medicine, New Orleans, LA USA; 3 Department of Orthopedics and Traumatology, University Hospital Queen Giovanna-ISUL, Medical University of Sofia, Sofia, 1527 Bulgaria; 4 Department of Neurosurgery, Tulane University School of Medicine, 131 S. Robertson St. Suite 1300, New Orleans, LA 70112 USA; 5 Department of Neurology, Tulane Center for Clinical Neurosciences, Tulane University School of Medicine, New Orleans, LA USA; 6 Department of Neurosurgery and Ochsner Neuroscience Institute, Ochsner Health System, New Orleans, LA USA; 7 Dental and Oral Medical Center, Kurume University School of Medicine, 67 Asahi-machi, Kurume, Fukuoka Japan; 8 Division of Gross and Clinical Anatomy, Department of Anatomy, Kurume University School of Medicine, 67 Asahi-machi, Kurume, Fukuoka Japan; 9 Department of Anatomical Sciences, St. George’s University, St. George’s, Grenada; 10 Department of Otolaryngology, Tulane University School of Medicine, New Orleans, LA USA; 11 Department of Surgery, Tulane University School of Medicine, New Orleans, LA USA; 12 University of Queensland, Brisbane, Australia

**Keywords:** Anatomy, Cubital tunnel, Peripheral nerve, Neuropathy, Upper limb

## Abstract

Objective Almost 70 years ago, Osborne described a fibrous band, Osborne’s ligament, stretching between the humeral and ulnar heads of the flexor carpi ulnaris, which forms the cubital tunnel’s roof. Prior to this account, this ligament had received limited attention in the anatomical literature and had been poorly studied. Therefore, the present anatomical study was performed to better elucidate this structure. Methods thirty adult anatomical donors (60 sides) underwent dissection of the medial aspect of the elbow with a focus on Osborne’s ligament. Identified ligaments were documented, classified, and measured. Additionally, histological evaluation of these structures was performed. Results osborne’s ligaments were identified on 49 sides (81.7%). These were classified as absent (type I; 18.3%), wide and thin and more distally located (type II; 51%), narrow and more proximally located (type III: 39%), coexistent with an anconeus epitrochlearis muscle (type IV; 6%), and absent with only anconeus epitrochlearis muscle (type V; 4.1%). When coexistent with anconeus epitrochlearis muscle (6%), the anconeus epitrochlearis muscle was usually located more proximally, although in some cases, it was in the same plane, especially with larger anconeus epitrochlearis muscles. Conclusions based on our findings, the Osborne’s ligament is not a ligament but is usually made up of aponeurosis/tendon over the proximal flexor carpi ulnaris muscle and can coexist with the anconeus epitrochlearis muscle. This structure comes in various forms, illustrating that this term is not specific to a single structure.

**Clinical trial number**: Not applicable.

## Introduction

A thorough understanding of the elbow’s anatomy is essential for accurately diagnosing and managing related pathologies. In cases of ulnar nerve entrapment, the cubital tunnel plays a pivotal role in the disease mechanism, diagnosis, and surgical intervention [1–5].

**Fig. 1:**
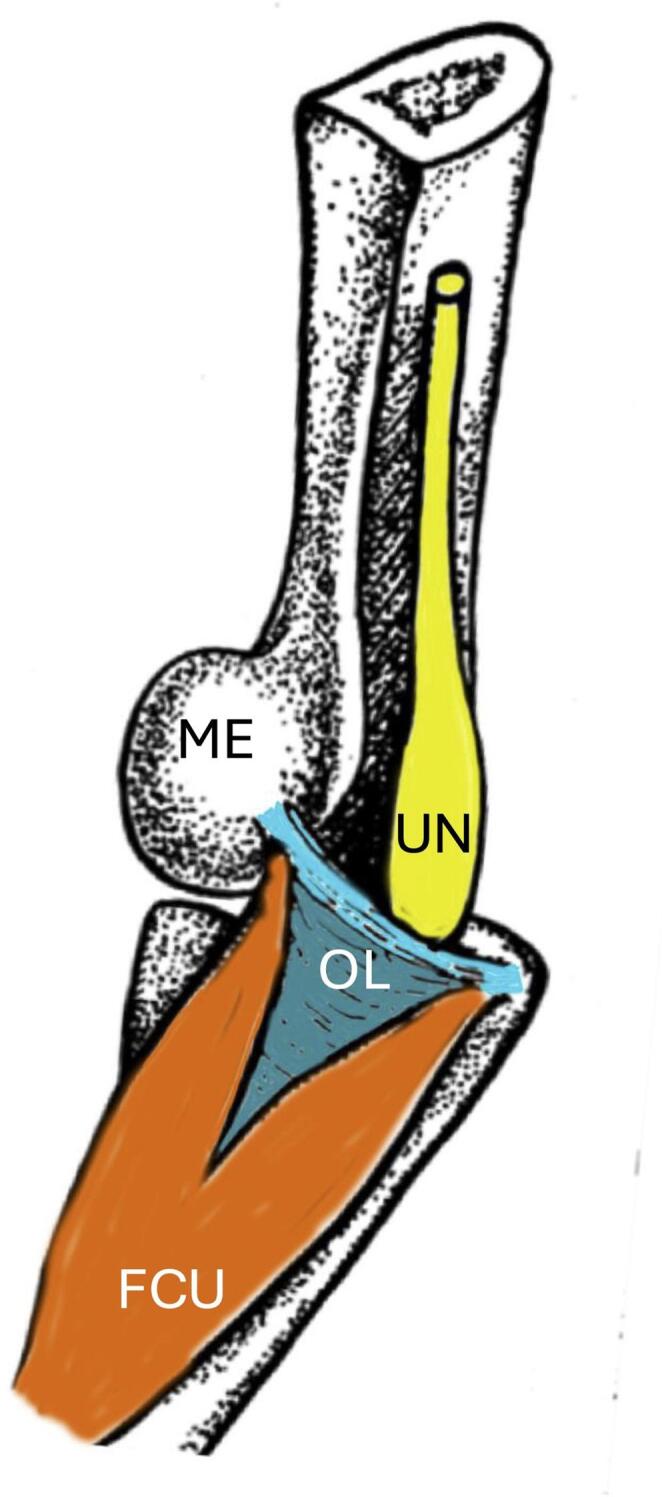
Schematic drawing of Osborne’s ligament (after Osborne *Postgraduate Medical Journal* 35, 392 − 96, 1959.) Note the ulnar nerve (UN) passing deep to Osborne’s ligament (OL) with two sets of fibers: proximal fibers (light teal) connecting the medial epicondyle of the humerus (ME) to the olecranon and more distal fibers (dark teal) uniting the two heads of the flexor carpi ulnaris muscle (FCU). ME, medial epicondyle of the humerus

In 1957, Geoffrey Vaughan Osborne, a British orthopaedic surgeon described a fibrous band stretching between the humeral and ulnar heads of the flexor carpi ulnaris, which forms the cubital tunnel’s roof (Fig. 1). This structure, known eponymously as Osborne’s ligament (OL) [6] has also been labeled the arcuate ligament of Osborne [7], cubital (tunnel) retinaculum [1], Osborne’s fascia [2], Osborne’s band [8], and tendinous arch [7].

OL is hypothesized to be a vestigial remnant or an evolved variant of the anconeus epitrochlearis muscle, [9] which may confer functional advantages in sustained elbow flexion [10]. In cases where the anconeus epitrochlearis is present, it may replace the ligament entirely [11]. Anatomically, the ligament attaches firmly to the medial epicondyle of the humerus while maintaining a more mobile connection to the olecranon [4]. It remains relaxed in elbow extension and becomes taut during elbow flexion, beginning to tighten at 135° and becoming well-defined at 90° [4].

Before Osborne’s account, this ligament received limited attention in the anatomical literature and has been poorly studied even today. Moreover, the exact definition, location, and composition of this structure are not agreed upon. Therefore, the purpose of this study was to clarify the detailed anatomical characteristics of this structure and to assess their clinical relevance (Table 1).

**Table 1:** Studies with reported prevalence of Osborne’s ligament

Study	Population studied	Number	Methods used	Reported OL prevalence
James et al., 2011	Cadaveric upper limbs	25 elbows	Dissection	8%
Dellon et al., 1986	Cadaveric upper limbs	104 upper limbs	Dissection	77%
O’Driscoll et al., 1991*	Cadaveric elbows	27 elbows	Dissection	96.3%
Karataş et al., 2009	Cadaveric upper limbs	20 elbows	Dissection	> 90%

* O’Driscoll et al. quantified the cubital tunnel retinaculum (*CTR*) rather than explicitly labeling the structure “Osborne’s ligament.” *CTR* presence is frequently treated as equivalent to the roof structure implicated in OL-related cubital tunnel compression

## Materials and methods

Thirty adult anatomical donors (60 sides) (17 males and 13 females) underwent dissection of the medial aspect of the elbow with a focus on OL. Skin and subcutaneous tissue were carefully removed to expose the muscular, fascial, or ligamentous tissue.

This cohort’s mean age at death was 80 years (65–101 years). The specimens were all formalin-fixed. Identified ligaments were documented, photographed, and classified, and random histological samples of the OL and its variations were evaluated using hematoxylin and eosin, Masson’s trichrome, and Van Gieson staining. Slides were viewed under a light microscope (EVOS FL Auto, Life Technologies, Carlsbad, CA, USA).

Statistical analyses comparing ligament presence and type between sides and sexes were performed using Fisher’s exact test, Chi-square test, and McNemar’s test in SPSS Statistics (version 29.0; IBM Corp., Armonk, NY, USA). A p-value of < 0.05 was considered statistically significant.

The authors state that every effort was made to follow all local and international ethical guidelines and laws that pertain to the use of human anatomical donors and their images in anatomical research.

## Results

### Gross anatomical findings

**Fig. 2:**
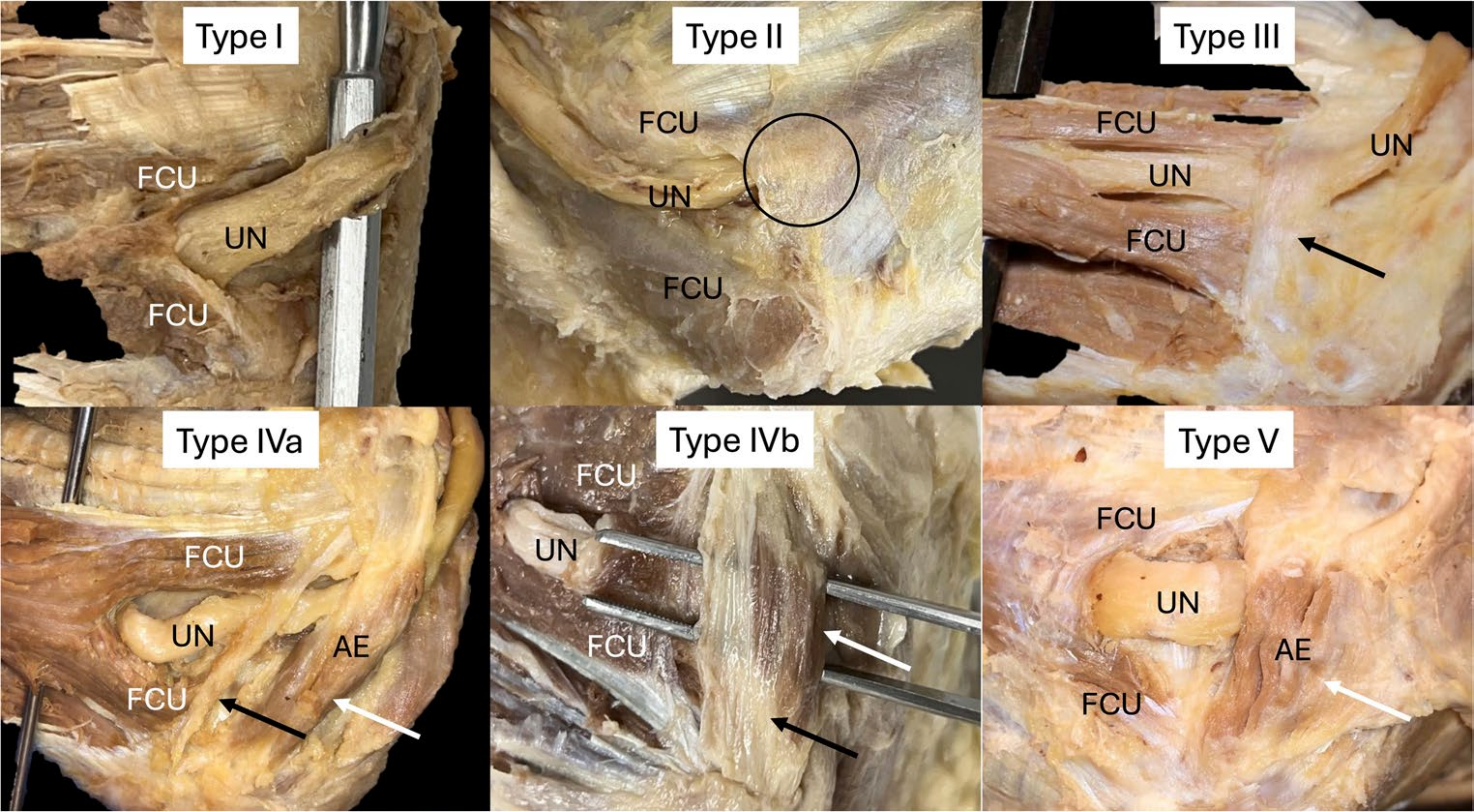
Cadaveric dissections noting gross anatomical variations of Osborne’s ligament (OL) and the classification used in the present study (types I-V). UN, ulnar nerve; AE, anconeus epitrochlearis muscle; FCU, flexor carpi ulnaris muscle. Type I is the absence of OL, type II is a thin and wide band-like OL (circle) between the two heads of flexor carpi ulnaris, type III is a thick and narrow ligamentous-like band OL (black arrow) traveling between the medial epicondyle of the humerus (ME) and olecranon, type IV shows coexistence of an OL (black arrow) and AE (white arrow), and type V is the absence of OL and only an AE (white arrow). Note the OL and AE are separate entities in type IVa but fused in type IVb

OLs were grossly identified on 49 sides (81.7% of sides). These were classified as absent with no AE (type I; 11/60; 18.3%), wide and thin and more distally located (type II; 25/49; 51.0% sides), narrow and more proximally located (type III; 19/49; 38.7%), coexistent with AE (type IV; 3/49; 6.1% sides), and absent with only AE (type V; 2/49; 4.1%). Type IV was further divided into two subgroups; the OL was separate from AE, which was located more proximally (type IVa; 4.1%), and the OL fused with larger AE, which was located more distally (type IVb; 2.1%) (Figure. 2).

### Histological findings

**Fig. 3:**
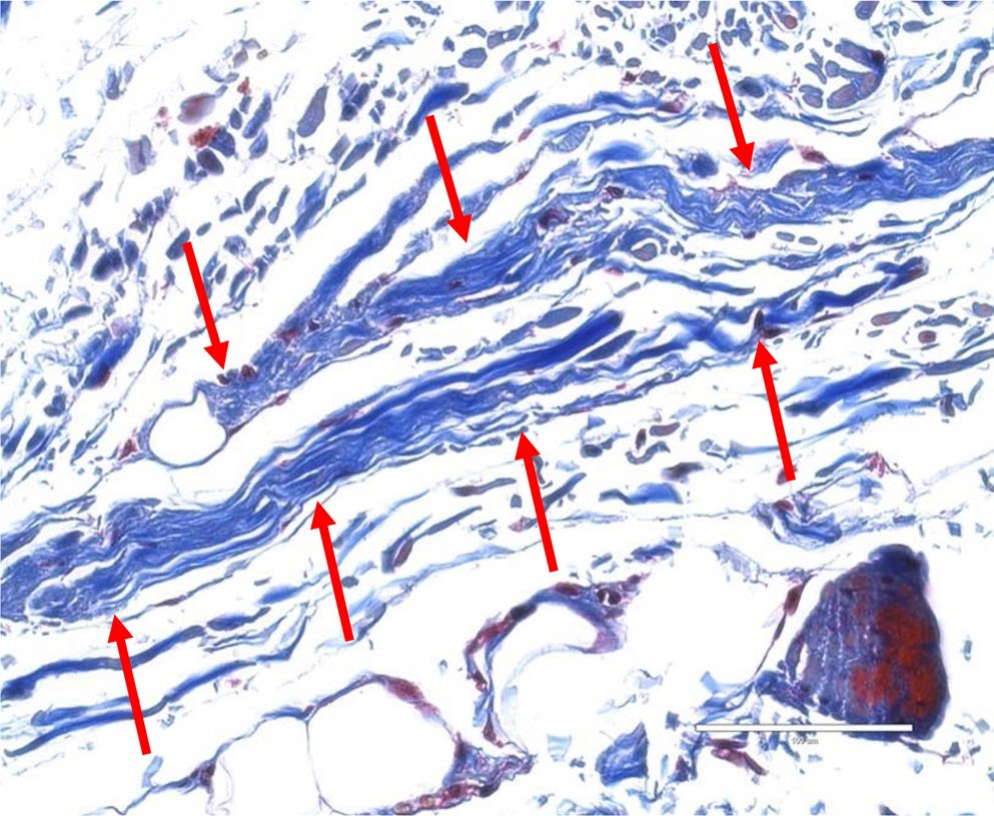
Histological views of type II (Masson’s trichrome). Note the irregularly arranged, wavy, blue-stained fibers indicative of collagen (arrows). There is an undulating, loosely organized pattern. Scale bar: 100µm

**Fig. 4:**
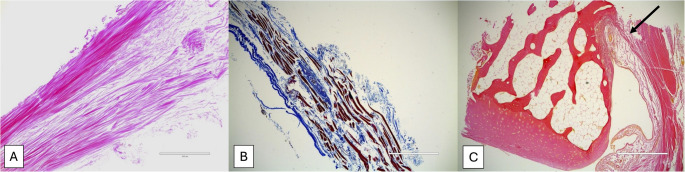
Histological views of type III. **A**: Hematoxylin and eosinstaining. Note the more uniform, highly organized, and parallel collagen fibers arranged in rows and the lack of elastic fibers. Scale bar, 400µm. **B**: Masson’s trichrome staining. Scattered skeletal muscle fibers with randomly positioned collagen fibers. Scale bar, 1000µm. **C**: Van Gieson staining. The far-right image shows attachment onto the medial epicondyle of the humerus and suggests a fibrocartilaginous enthesis (the enthesis is defined as the area where tendon, ligament, or joint capsule inserts into bone and acts to transmit tensile load from soft tissues to bone).20 The fibers are inserted directly into the bone, a hallmark of Sharpey’s fibers. Additionally, a faint transitional zone is seen between the uncalcified and subchondral bone regions, indicative of a tidemark seen at tendinous attachments into bone. Scale bar, 1000µm.

**Fig. 5:**
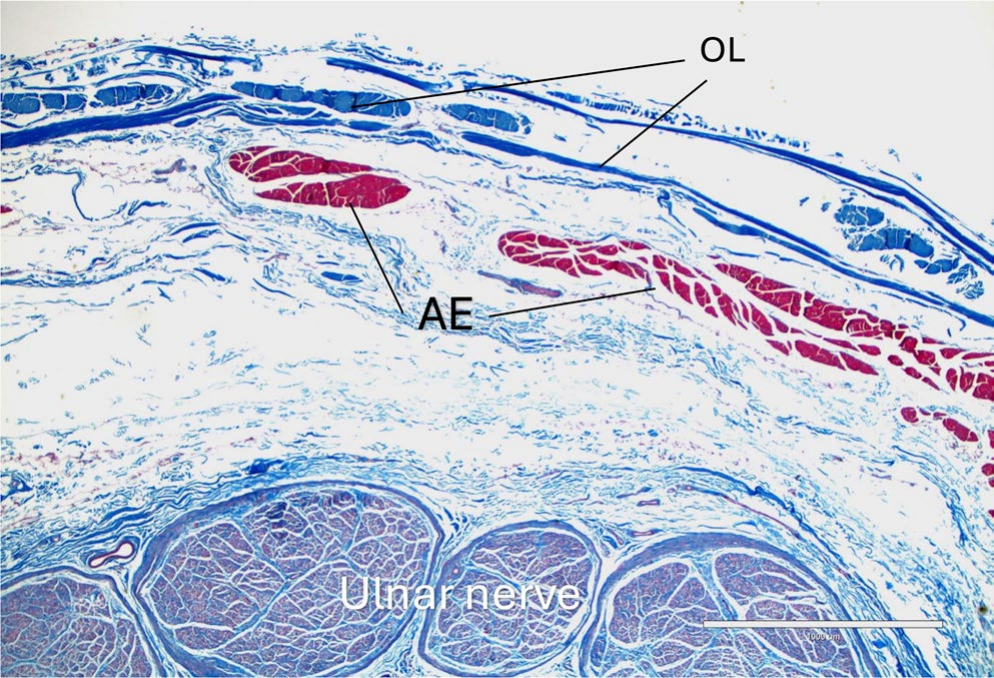
Histological view of type IV (Masson’s trichrome staining). Note the coexistence of both tendinous and muscular components. Compare to Figure 2. Scale bar: 1000µm

**Fig. 6:**
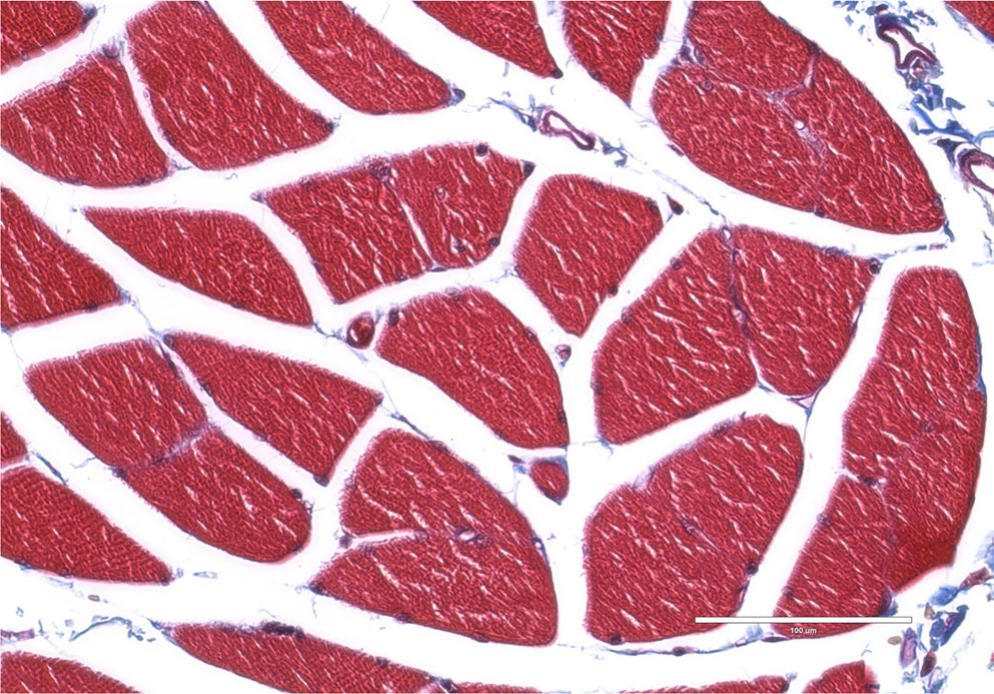
Histological view of type V (Masson’s trichrome staining). Note the tissue is consistent with normal skeletal muscle. Scale bar: 1000µm

Histological samples were randomly selected from each type except for type I. The connective tissue forming the roof of the cubital tunnel, i.e., OL, was histologically, thin band-like collagen fibers (type II, figure 3), uniform, highly organized, and parallel collagen fibers without elastic fiber or scattered skeletal muscle fibers with randomly positioned collagen fibers (type III, figure 4), tendon with interposition of skeletal muscle fibers (type IV, Figure. 5), or skeletal muscle fibers (type V, Figure 6).

Based on the gross anatomical and histological findings, we propose the following classification of Osborne’s ligament (OL):

- Type I:  Absence of OL and absence of AE (Figure 1)
- Type II: Fascial OL (wide and thin band), possibly a component of the deep antebrachial fascia (Figures 2 and 3)
- Type III: Thickened (Cord-like) OL (narrow and thick band), with or without AE embedded within it (Figures 2 and 4)
- Type IV: Tendinous OL, with either a separate AE (type IVa) or a fused AE (type IVb) (Figures 2 and 5)
- Type V: Absence of OL with presence of AE (Figures 2 and 6)

Type II was considered a component of the deep antebrachial fascia, lacking any tendinous or muscular elements. In contrast, types III, IV, and V consistently contained tendinous or skeletal muscle tissue, with histological examination revealing the presence of skeletal muscle fibers in the majority of these cases. Based on these findings, we concluded that type II represents a false Osborne’s ligament (OL), while types III, IV, and V constitute true OL. Furthermore, the tendinous or muscular structure forming the roof of the cubital tunnel, which historically has been referred to as “Osborne’s ligament,” is more accurately identified as the anconeus epitrochlearis muscle, whether as a remnant, degenerated form, or distinct anatomical entity.

No specimen was found to have signs of previous trauma to the elbow or findings consistent with ulnar nerve compression at the elbow, e.g., atrophy of flexor carpi ulnaris or hypothenar muscles.

Although the anconeus epitrochlearis muscle was found slightly more commonly on the right side, this did not reach significance. Statistical comparison of OL presence between sexes showed no significant difference (*p* ≥ 0.05, Fisher’s exact test). Comparison of OL presence between right and left sides showed no significant side preference (*p* ≥ 0.05, McNemar’s test). The distribution of OL types between sexes was not statistically significant (*p* ≥ 0.05, chi-square test).

## Discussion

Our study found that OL is not a true ligament but rather a structure composed of fascia, tendon (or aponeurosis), or skeletal muscle. Therefore, the term “Osborne’s ligament” may be anatomically inappropriate. However, for consistency with existing literature, we continue to use the term ‘OL’ in the discussion below. We suggest a new classification: cubital tunnel roof types I-V. This better represents the gross and microscopic anatomy and variations of OL and will aid in communication, both surgically and for future studies. Also, the more proximal OL contained skeletal muscle on histology and probably represents a degenerated AE. Therefore, the prevalence of AE in the literature, based on imaging and gross dissection, is underestimated.

Morphometric analyses have shown that OL has a mean thickness of approximately 0.15–0.18 mm and spans about 2.2 cm in length with a width of roughly 4 mm [1, 6, 9, 12]. Magnetic resonance imaging studies have identified OL thickening (≧ 2 mm) in 8% of individuals [7]. In some disorders of the connective tissues, such as Ehlers-Danlos syndrome, increased ligamentous laxity has also been observed [13].

A morphological classification proposed by O’Driscoll et al. categorized OL into four types based on gross anatomical dissection.

- Type 0: Absent.
- Type Ia: Lax in extension, taut in full flexion.
- Type Ib: Taut prior to full flexion (typically 90–120°).
- Type II: Replaced by the anconeus epitrochlearis.

Reported prevalence rates of OL vary widely. Some anatomical studies have found it in over 90% of specimens [1], while others report rates as low as 8% [12]. This discrepancy likely reflects differences in definitions, dissection techniques, and population variance [10, 14]. Based on our anatomical findings, such data should be questioned, as the OL was very variable, not ligamentous, and histologically, it was often mixed with skeletal muscle fibers.

### Clinical and surgical relevance

The ulnar nerve is the most commonly compressed nerve at the elbow, and OL has frequently been implicated in such entrapment syndromes.[3–5, 8, 15–18] With increased elbow flexion, some have found that the volume decreases deep to OL in the cubital tunnel, potentially exacerbating nerve compression [1]. Silva et al. [19] reported that the mean length of the OL was 29.6 mm in flexion and 18.9 mm in extension. The measurement difference supports the idea that elbow flexion increases tension in the OL, which may contribute to ulnar nerve compression in the cubital tunnel. Osborne himself reported symptom relief in numerous patients following the surgical division of OL, highlighting its clinical significance [4, 15]. Notably, over 10% of his patients with idiopathic ulnar neuritis showed improvement following this decompressive procedure [4]. Interestingly, in the illustration used by Osborne (Fig. 1), this band of tissue was shown as having two parts: a proximal thin part uniting the medial epicondyle to the olecranon and a wide, more distal part uniting the humeral and ulnar heads of the flexor carpi ulnaris muscle. This suggests that the original description of the band included two separate structures.

## Conclusion

Based on our findings, the OL is not a ligament but usually comprises aponeurosis/tendon over the proximal FCU and can coexist with the AE. It can contain sparse skeletal muscle fibers whether or not it coexists with the AE. This structure comes in various forms, illustrating that this term is not specific to a single structure. Therefore, we suggest that the term OL not be used, but cubital tunnel roof types I-V, which will decrease confusion in future medical communication. Such knowledge will also be important to surgeons decompressing the ulnar nerve at the cubital tunnel so that the variations of the cubital tunnel roofs are better understood. The preoperative ultrasound examination could be useful to identify the existence of the AE.

### Limitations

Results may be different between symptomatic patients and anatomical donors.

## Data Availability

No datasets were generated or analysed during the current study.
